# Supplementary material for: Martian ionospheric response during the May 2024 solar superstorm
Source: Nat Commun. 2026 Mar 5;17:2017. doi: 10.1038/s41467-026-69468-z (PMC12963534; doi:10.1038/s41467-026-69468-z)
Supplement: Supplementary file 1 — Supplementary Information [file 41467_2026_69468_MOESM1_ESM.pdf]

Supplementary Materials for

Martian Ionospheric Response during the May 2024 Solar Superstorm.

Jacob Parrott, Beatriz Sánchez-Cano, Håkan Svedhem, Olivier Witasse, Dikshita Meggi, Colin Wilson, Alejandro Cardesin-Moinelo, Ingo Müller-Wodarg
Corresponding author: Jacob.parrott@esa.int

The PDF file includes:

Supplementary Figures 1 to 6

Supplementary Figure 1

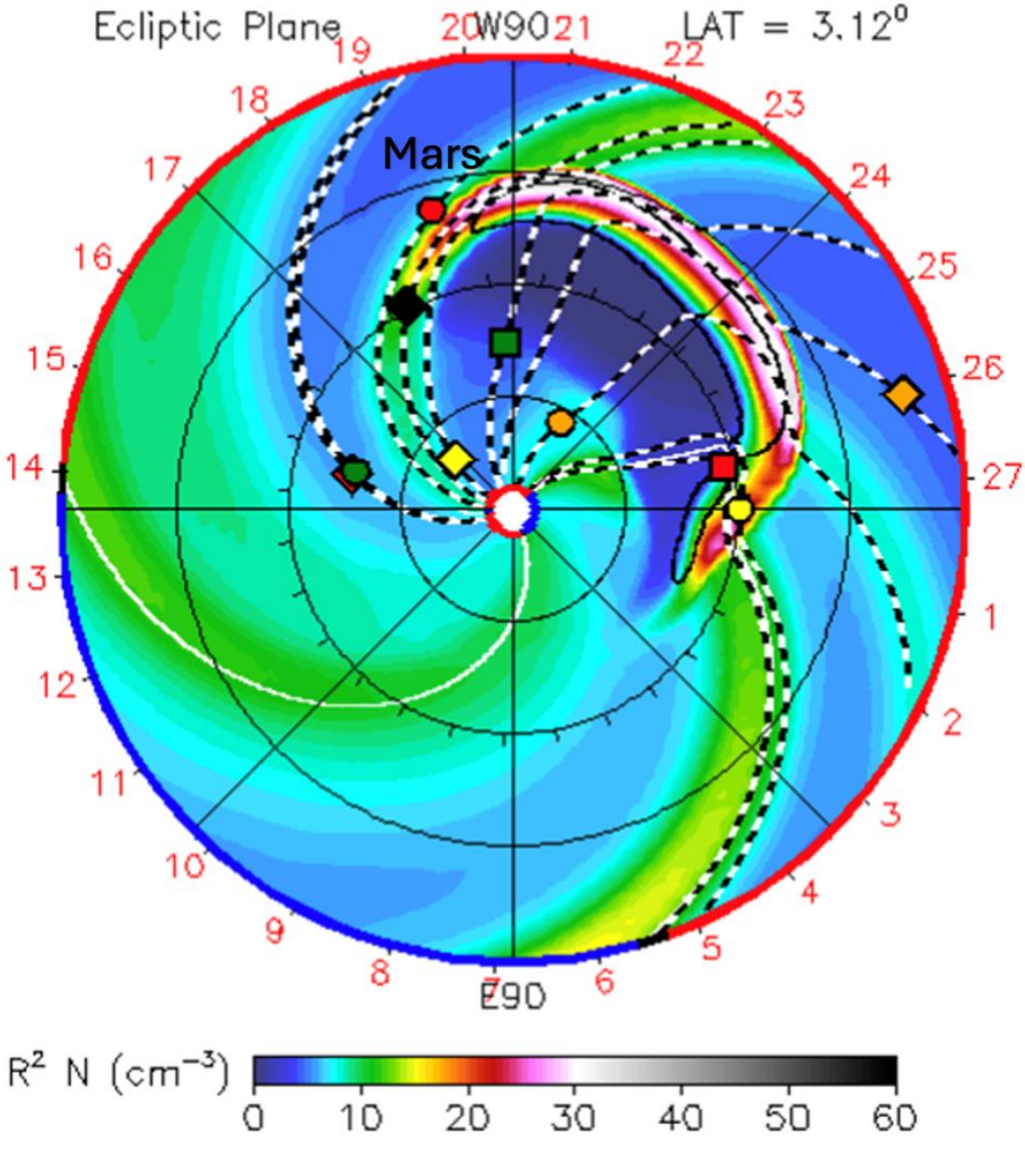

Caption: The results of a Wang-Sheeley-Arge simulation in the solar polar coordinate reference frame by the Moon to Mars (M2M) office for the hour that the coronal mass ejection impacted Mars. This figure shows that the semi-halo CME is impacting mars very close to when the mutual radio occultation measurement took place. Interplanetary magnetic field shown in zebra lines, colours correspond to particle density, circle markers indicated planets (orange is Mercury,

green is Venus, yellow is Earth and red is Mars) and square markers represent solar monitoring spacecraft (yellow is BepiColombo, green is Parker Solar Probe, black is OSIRIS-APEX and orange is Lucy.) The exact output for this simulation can be found at: https://kauai.ccmc.gsfc.nasa.gov/DONKI/view/WSA-ENLIL/30726/1

This figure illustrates the geometric configuration of Mars in relation to the Coronal Mass Ejection (CME). The red circle denotes the position of Mars, while the yellow circle represents the position of Earth. The red arc emanating from the origin signifies the mass ejection ion density, clearly indicating that Mars was impacted by the CME on May 15, 2024. Whilst we are showing a single simulation here, we appreciate that atmospheric heating can be caused by the compounding effects of multiple CMEs. So please follow this link to find a selection of CMEs from around the time of our mutual radio occultation measurement. Datasets for this time period are is available at: https://kauai.ccmc.gsfc.nasa.gov/DONKI/search/results?startDate=2024-05-10&endDate=2024-05-15&catalog=M2M_CATALOG&eventType=CME

Supplementary Figure 2

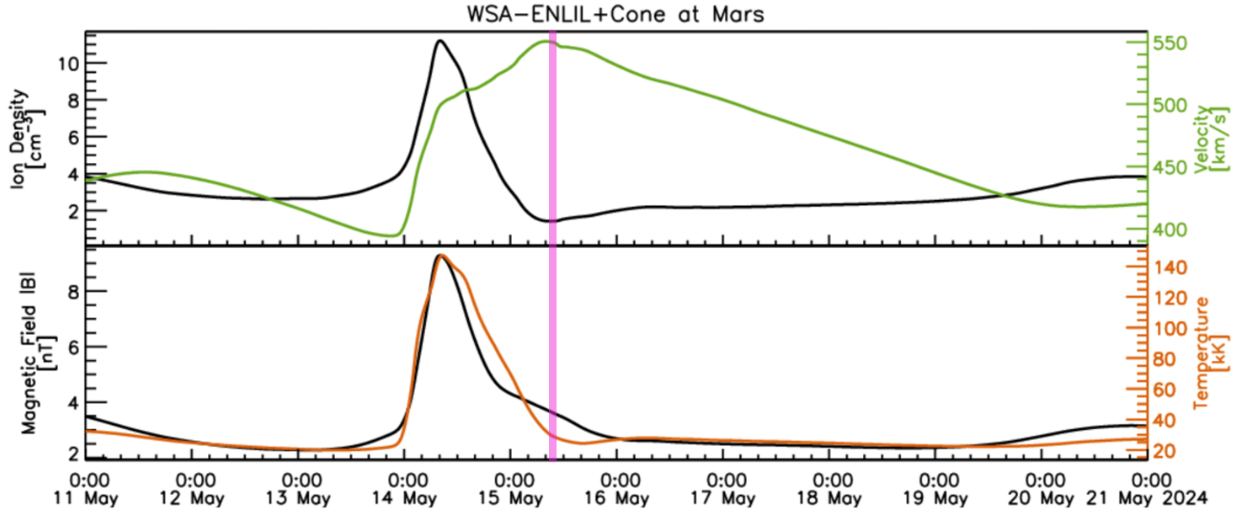

Caption: The results of a Wang-Sheeley-Arge simulation for a solar disturbance at Mars by the M2M office, showing the ion density, velocity and magnetic field strength and temperature of the coronal mass ejection. A magenta line indicates the moment of the mutual radio occultation measurement. The exact output for this simulation can be found at: https://kauai.ccmc.gsfc.nasa.gov/DONKI/view/WSA-ENLIL/30726/1

The moment of the mutual radio occultation measurement coincided with the solar flare's intensity rising toward its peak, accompanied by an associated increase in solar energetic particle (SEP) flux.

Supplementary Figure 3

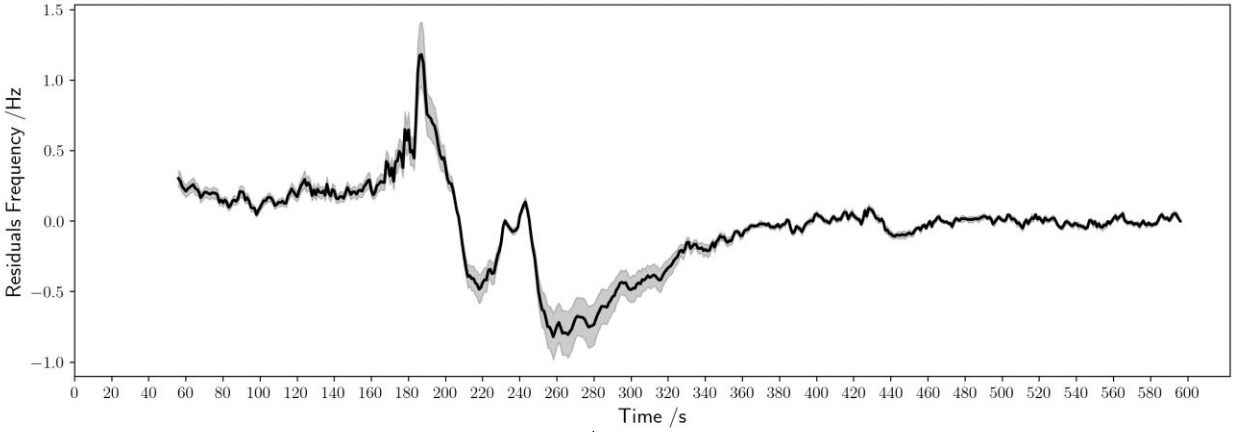

Caption: The residuum for the profile from 15th of May 2024. This is the residual frequency shift that is produced as the UHF radio link passes through the Martian ionosphere and atmosphere. The grey envelope is a 1σ uncertainty.

Fig S3 is the minute frequency shift that occurs when the 437.1 MHz radio link between MEX and TGO penetrates the ionosphere and atmosphere at different altitudes. This measurement was an egress occultation, which means the measurement began with the line-of-sight between the two spacecraft being intercepted by the Martain surface, then as the measurement progresses, the radio link raises altitude until it is passing only through the vacuum of space. This can be seen in this figure as the frequency deviations can be seen at the beginning of the measurement, and then it tapers down to 0 Hz after 340 s. The feature between 180 and 240 s is due to the large M1 ionospheric feature, and the feature between 240 and 340 s is from the M2.

An in-depth explanation for the conversion of this residuum to a vertical electron density profile can be found in Parrott et al., 2024 [1] and the technique is described in the Methods section.

The uncertainty for this has been calculated via the same method used in Parrott et al., 2024 (where it is explained in greater depth). This involves the addition of a 5% magnitude noise being added to an earlier stage of the processing chain, when the Doppler shift due to the relative motion of the spacecraft still dominates the signal. Figure S3 shows that the uncertainty grows around the M1 and M2 ionospheric features between 160 and 340 s. This is because a step in the processing chain involves a model fit to the oscillator drift during the vacuum portion of the signal (here, this is after 360 s), meaning the uncertainty is mostly removed for this section. A similar thing happens for a point just under the M1 layer, where the net refractivity between the neutral atmosphere and the ionosphere balances. This is the same method used to find the uncertainty for Figure 2 and 3.

Supplementary Figures 4

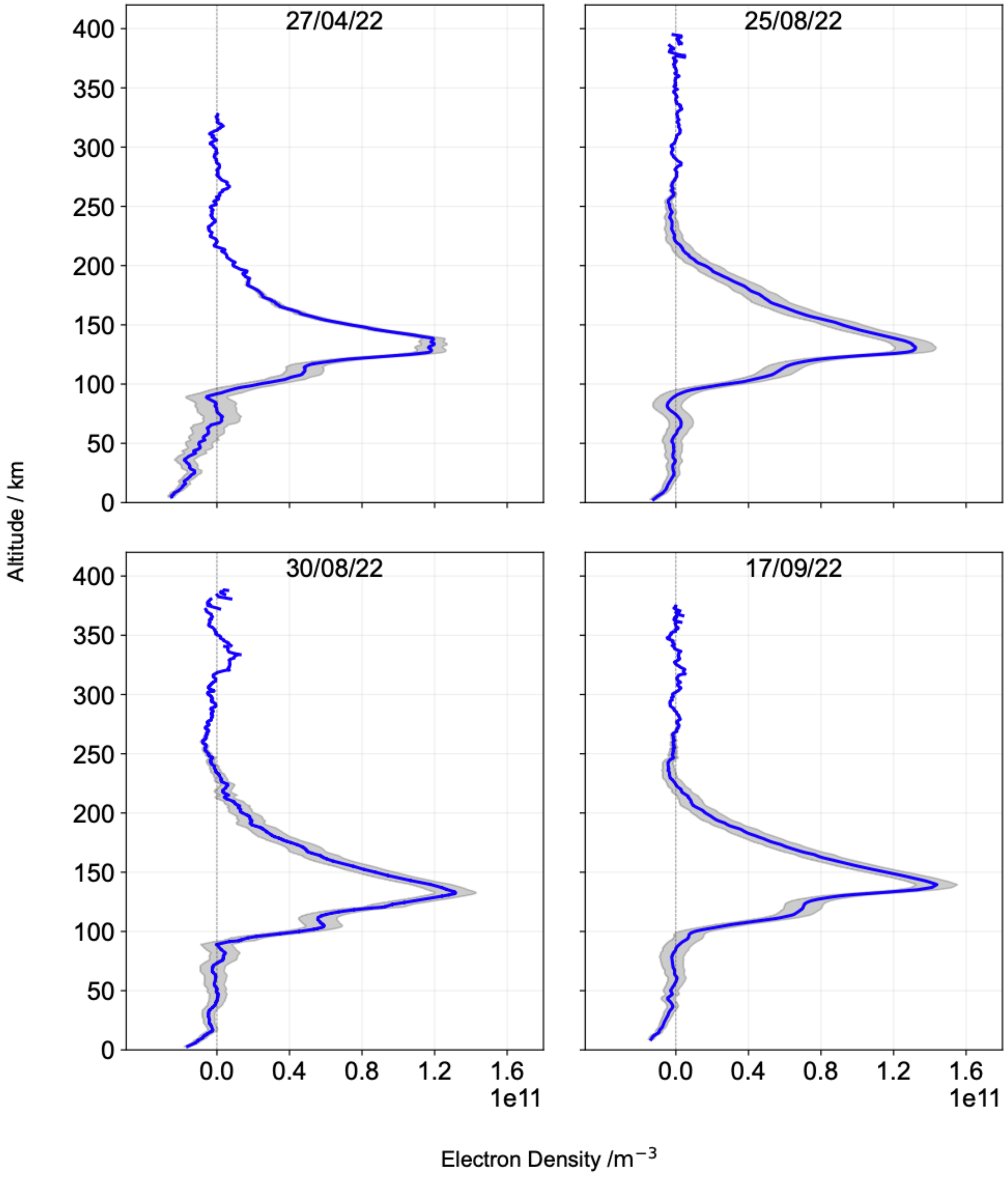

Caption: 12 comparable electron density profiles from measurements with comparable SZA values (48° - 58°), along with the solar storm effected measurement from 15/05/24 to 17/09/22. These are the same profiles shown in Figure 2, but plotted separately for clarity. Error bars have been calculated in the same way described in S3. They show how large the M1 and M2 ionospheric layers are compared to other vertical profiles captured from similar solar zenith angles.

Supplementary Figures 5

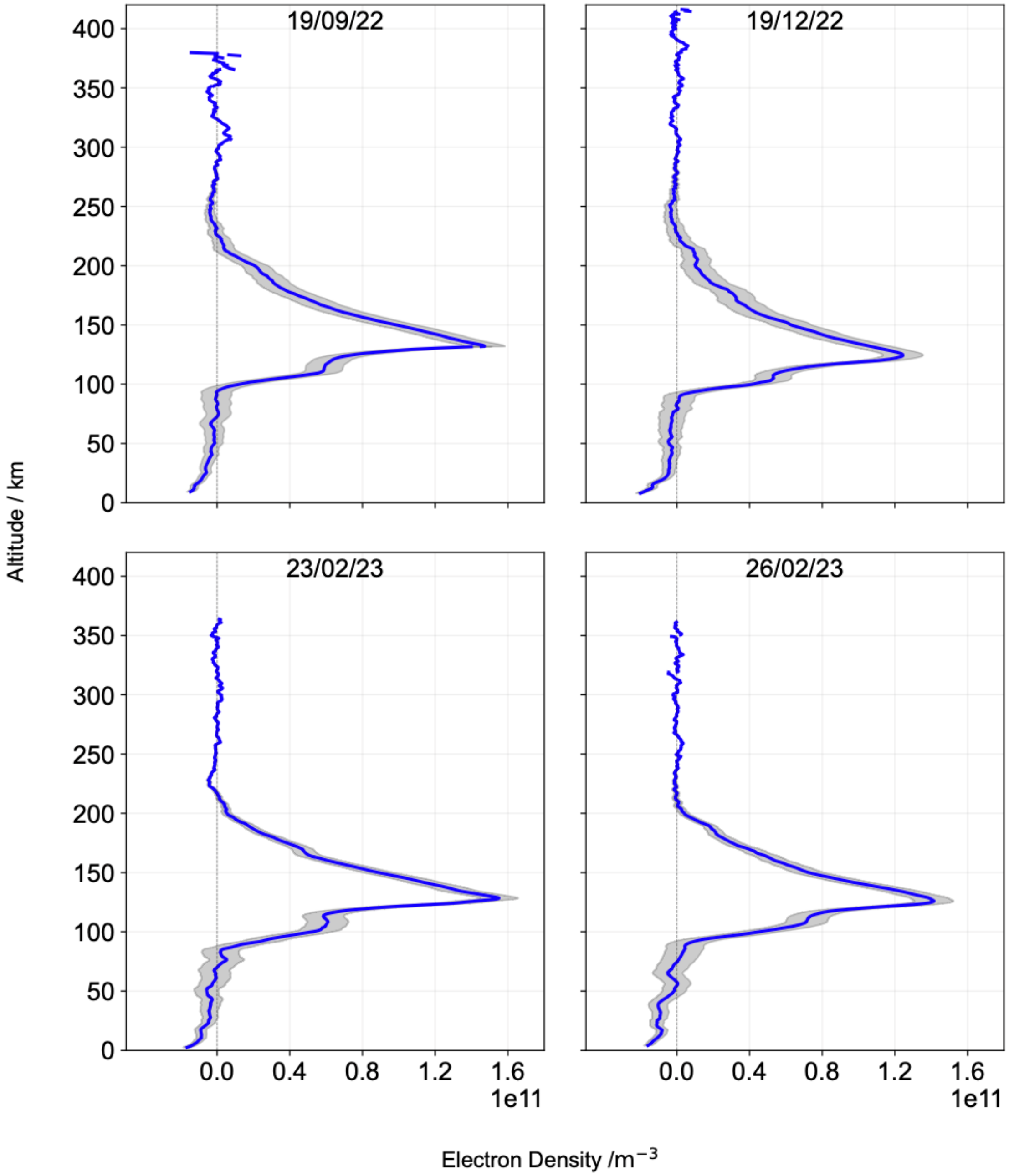

Caption: 12 comparable electron density profiles from measurements with comparable SZA values (48° - 58°), along with the solar storm effected measurement from 19/09/22 to 26/02/23. These are the same profiles shown in Figure 2, but plotted separately for clarity. Error bars have been calculated in the same way described in S3. They show how large the M1 and M2 ionospheric layers are compared to other vertical profiles captured from similar solar zenith angles.

Supplementary Figures 6

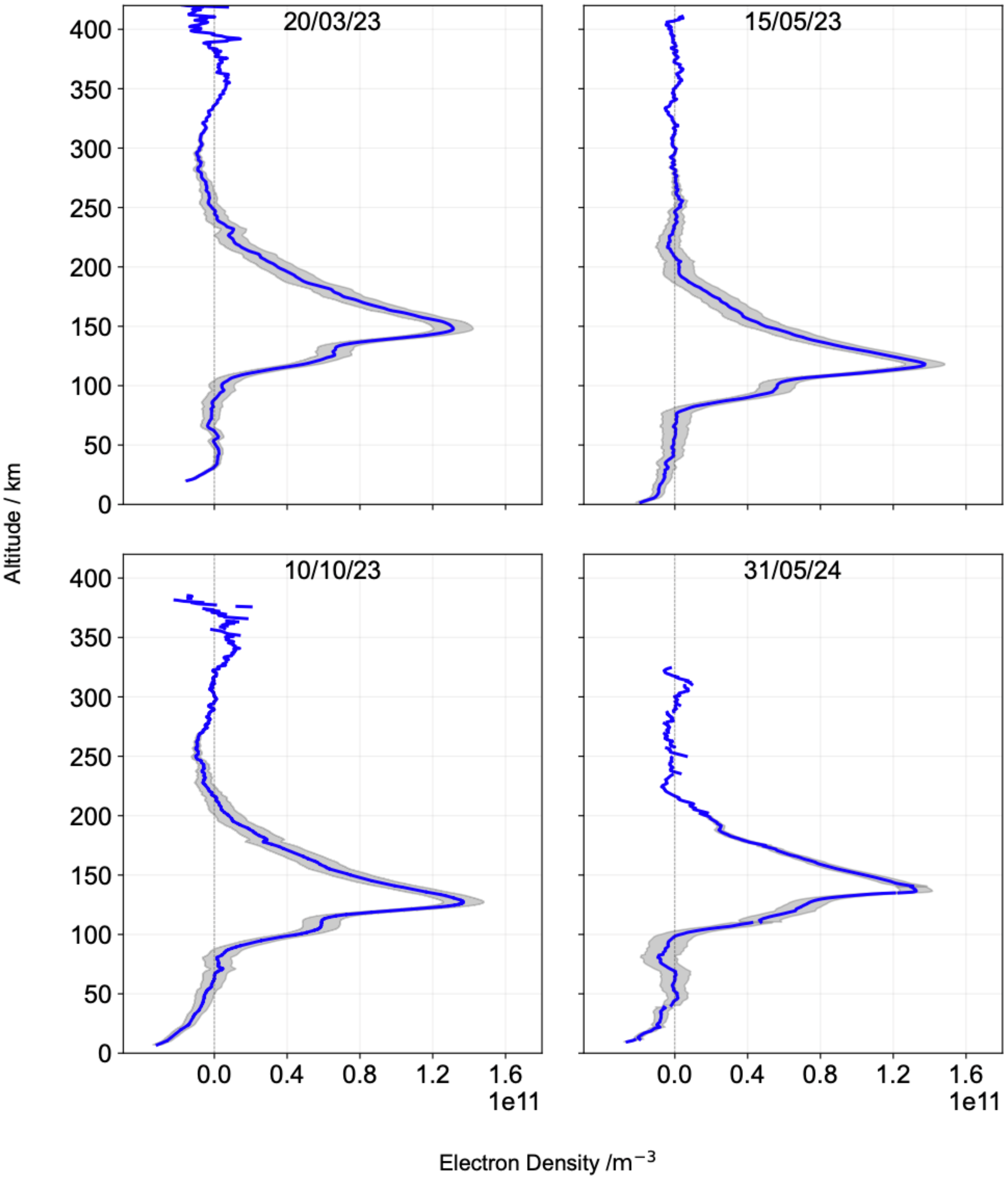

Caption: 12 comparable electron density profiles from measurements with comparable SZA values (48° - 58°), along with the solar storm effected measurement from 20/03/23 to 31/05/24. These are the same profiles shown in Figure 2, but plotted separately for clarity. Error bars have been calculated in the same way described in S3. They show how large the M1 and M2 ionospheric layers are compared to other vertical profiles captured from similar solar zenith angles.

References

[1] J. Parrott *et al.*, 'First Results of Mars Express—ExoMars Trace Gas Orbiter Mutual Radio Occultation', *Radio Sci.*, vol. 59, no. 7, p. e2023RS007873, Jul. 2024, doi: 10.1029/2023RS007873.
